# Sitting on the sidelines: Disparities in social, recreational, and community participation among adolescents with Autism Spectrum Disorder

**DOI:** 10.1007/s10803-021-05216-0

**Published:** 2021-07-31

**Authors:** Alexa C. Budavari, Elise T. Pas, Gazi F. Azad, Heather E. Volk

**Affiliations:** 1 Department of Mental Health, Johns Hopkins Bloomberg School of Public Health; 2 Division of Child and Adolescent Psychiatry, Department of Psychiatry, New York State Psychiatric Institute and Columbia University, Center for Autism and the Developing Brain, Weill Cornell Medicine; 3 Wendy Klag Center for Autism and Developmental Disabilities, Johns Hopkins Bloomberg School of Public Health

**Keywords:** Autism Spectrum Disorder, sociodemographic disparities, extracurricular participation, community involvement, National Survey of Children’s Health

## Abstract

Participation in extracurricular activities and community involvement during secondary school is important for the healthy social, emotional, mental, and physical development of adolescents, especially those with autism spectrum disorder (ASD). The current study utilized three waves of data (2016, 2017, and 2018) from the National Survey of Children’s Health (NSCH) to examine disparities in extracurricular participation among 12- to 17-year old adolescents with ASD. Across the three waves, data demonstrate clear sociodemographic disparities among adolescents with ASD. These disparities were more evident in adolescents with caregivers that had less education and lower household income, as well as males. These disparities suggest a continued need for targeted interventions to promote engagement among adolescents with ASD to narrow this social disparity gap.

The World Health Organization (WHO) identifies social participation as a way to promote the healthy development and well-being of children (World Health Organization, 2007). Participation in extracurricular and community activities is critical to promoting mental wellness, physical health, and overall life satisfaction (Hilton, Crouch, & Israel, 2008; King et al., 2003; Murphy & Carbone, 2008; Potvin et al., 2013). Participation may be particularly important for individuals with autism spectrum disorder (ASD) as it helps to address social, emotional, and behavioral impairments by providing an opportunity to interact with peer models, learn emotion regulation skills, engage in physical activity, and navigate social situations (Fredricks & Simpkins, 2013; Hilton, Crouch, & Israel, 2008; Murphy & Carbone, 2008). Despite these potential benefits, numerous studies have shown that adolescents with ASD have significantly decreased levels of participation compared to their typically developing peers and adolescents with other disabilities (Askari et al., 2015; Taheri et al., 2016; Shattuck et al., 2011). Furthermore, rates of participation have been shown to significantly decrease from adolescence to young adulthood (Lounds Taylor, Adam, & Bishop, 2017; Myers et al., 2015; Simpson, et al., 2019). This diminished participation in young adults with ASD is evident in the high rates of social isolation, as well as low rates of employment and secondary education (Eilenberg et al., 2019; Roux et al., 2015). As a result, it is important to identify those adolescents with ASD who may already be experiencing low levels of social, recreational, and community participation before they experience further isolation and social disconnection as young adults.

Participation in extracurricular activities provides naturalistic opportunities for adolescents with ASD to address core impairments, including cognitive, behavioral, social, and emotional deficits (American Psychiatric Association, 2013). For example, participation in activities, such as clubs and organizations, provides an opportunity to learn about societal expectations, develop initiative, increase academic engagement, and learn emotion regulation (Bohnert, Lieb & Arola, 2019; Hilton, Crouch, & Israel, 2008; Denault & Poulin, 2009; Fredricks & Eccles, 2008). Given that adolescents with ASD tend to have limited peer relationships and face difficulties navigating social situations (Orsmond, Krauss, & Seltzer, 2004; Solish, Perry, & Minnes, 2010), these activities could be beneficial by providing an opportunity to connect with peers over shared interests, and, in turn, help to establish lasting friendships and buffer against future social isolation (Bohnert, Lieb & Arola, 2019). Additionally, participation in other structured activities, such as organized sports, provides the opportunity to work towards a shared goal through team work, increase physical activity, and boost self-esteem (Grandisson, Tétreault, & Freeman, 2012; Larson et al., 2006; McCoy & Morgan, 2020).

Community involvement, such as working for pay or engaging in community service, also provides multiple opportunities for growth and is linked to future success. Working for pay teaches time management skills, helps adolescents develop a vocational skillset, improves academic and career outcomes, and creates continuity through the post-high school transition (Hall, 2006; Mortimer, 2010; Musick & Wilson, 2008; Roux et al., 2015; Smith et al., 2010; Staff & Mortimer, 2007). Further, 90% of those individuals with ASD who had a job in high school continued to be employed after graduating, while only 40% of individuals who did not have a job in high school were employed as young adults (Roux et al., 2015). Therefore, engaging in work for pay while students are still in high school may be of higher importance for youth with ASD compared to typically developing peers given the increased accessibility of services provided through the school, such as those included in an Individualized Education Plan. Similarly, participating in community service provides multiple benefits including developing a skillset, increasing civic responsibility, along with increasing academic and career development (Hall, 2006; Musick & Wilson, 2008; Smith et al., 2010). Learning these skills in middle and high school, a significant period for socio-emotional development, can help improve functioning while adolescents are still in school and simultaneously provide future benefits by teaching vocational skills and connecting individuals to a community.

Unfortunately, adolescents with ASD miss out on the numerous benefits of participation. Compared to their typically developing peers, adolescents with ASD participate in less diverse activities with fewer participants, engage in fewer social, recreational, and vocational activities, and are less involved in the activities when they do participate (Egilson et al., 2017; Hilton, Crouch, & Israel, 2008; Potvin et al., 2013; Ratcliff et al., 2018; Solish, Perry, & Minnes, 2010; Taheri et al., 2016). Compared to adolescents with other disabilities (e.g.., intellectual disability, learning disability), adolescents with ASD engaged less frequently and were less involved in social and community activities (Shattuck et al., 2011; Taheri et al., 2016; Tint, Maughan, & Weiss, 2017).

Characteristics of the ASD phenotype itself, including social, cognitive, and behavioral impairments, serve as barriers to engagement in extracurricular and community activities (Dovgan et al., 2019; Ghanouni et al., 2019; Grandisson et al., 2012). Although these individual level factors serve as significant barriers, it is also necessary to consider the larger context in which these activities occur in order to develop targeted interventions that improve the accessibility and inclusiveness for adolescents with ASD. Caregivers, teachers, and self-advocates have already identified many environmental barriers to participation including a lack of suitable and accommodating activities, cost, peer rejection, and adult and staff attitudes (e.g., lack of understanding from activity leaders; Askarai et al., 2015; Egilson et al., 2017; Ghanouni et al., 2019; Krieger et al., 2018; Mattinson et al., 2018). In contrast, facilitators of participation include accommodating activities, trained staff, having a case manager to coordinate activities, transportation, social supports (e.g., existing friendships, parental support), and school services (Askari et al., 2015; Dovgan et al., 2019; Krieger et al., 2018; Myers et al., 2015; Pence & Dymond, 2015).

While previous studies have focused primarily on environmental barriers and facilitators to participation, to our knowledge, studies examining sociodemographic factors (e.g., race/ethnicity, SES), have been limited and shown mixed results. Research from the National Longitudinal Transition Study 2 (NLTS2), a national cohort study of adolescents receiving special education services, found that adolescents with ASD living in households in the lowest socioeconomic status (SES) category (i.e., < $25K/Year) were less likely to participate in any extracurricular activity (e.g., out of school activities, volunteering, lessons/classes) than adolescents in the highest SES category (> $75K/Year; Shattuck et al., 2011). In the same study, no differences in engagement in any extracurricular activity were found based on race, ethnicity, or gender. When examining only out of school activities, Hispanic adolescents with ASD had significantly lower levels of participation than White adolescents with ASD (Myers et al., 2015). Similar disparities in participation have also been found in neurotypical adolescents. For example, among secondary school students, adolescents who are not White, low SES, and/or female had lower rates of participation in sports (Im et al., 2016; Johnston, Delva, & O’Malley, 2007; Meier et al., 2018). In contrast, adolescents whose parents had low educational attainment more frequently engaged in work for pay during high school (Mortimer, 2010). Examining disparities based on SES and race/ethnicity in adolescents with ASD is particularly important given that young adults with ASD who are racial/ethnic minorities and/or live in low SES households have significantly lower rates of social participation, secondary education, and employment compared to young adults with ASD who are White and live in a higher income household (Eilenberg et al., 2019).

Previous work has also shown mixed results in younger samples of children with ASD. Among elementary and middle school-aged children with ASD, there were no gender differences for participation in community, neighborhood, or faith-based activities (Little et al., 2014). In the same sample, caregiver education was significantly associated with increased neighborhood activity participation only. Among school-aged youth with high-functioning autism, levels of community participation did not differ based on the child’s gender or parent education (Egilson et al., 2017). Although many other studies have included information on these sociodemographic factors, there has been limited exploration into whether they influence rates of participation among adolescents with ASD. In addition, there has been limited examination of the impact of sociodemographic factors on engagement in individual activities, as previous work has primarily focused on combined categories of participation. It is necessary to further explore whether sociodemographic factors influence participation in specific extracurricular and community activities among middle and high school youth with ASD. Such work will enable us to better identify those adolescents who already demonstrate decreased social and community engagement, which may have otherwise gone unnoticed when looking at combined rates of participation.

## Current Study

The current study sought to examine patterns of participation in extracurricular activities and community involvement among adolescents with ASD, stratified by sociodemographic factors, using data from the National Survey of Children’s Health (NSCH). Given the mixed results of previous studies examining sociodemographic disparities, we sought to identify those adolescents at the greatest risk of social and community disconnection by identifying sociodemographic factors associated with specific social, recreational, and community activities among adolescents with ASD. We utilized the most recent waves of data (i.e., 2016, 2017, and 2018) from the NSCH to examine whether sociodemographic disparities existed among adolescents with ASD based on sex, household income, or caregiver education in their engagement in sports, clubs, organized activities, work for pay, and community service.

## Method

### Participants

The study sample was comprised of the three most recent waves of the National Survey of Children’s Health (NSCH; Child and Adolescent Health Measurement Initiative (CAHMI), 2016, 2017, and 2018). The NSCH aims to produce state and national prevalence estimates for health indicators for non-institutionalized children aged 0 to 17. The survey is funded by the U.S. Health Resources Service Administration (HRSA) Maternal and Child Health Bureau (MCHB), and it is administered by the National Center for Health Statistics at the Centers for Disease Control and Prevention. Further information about survey methodology can be accessed online (CAHMI, 2017; 2019). The NSCH is a population-based, cross-sectional survey completed by caregivers about numerous health indicators for the selected child. Caregivers were surveyed via mail or web-based survey. Households from all 50 U.S. states were identified by address-based sampling, which identifies a random subset of houses from a list of residential addresses, conducted by the Census Bureau. From these identified households, families with one or more children under the age of 18 were identified via random mailings. For those caregivers that agreed to participate, one child was selected from the household to be the subject of the survey. Among families with multiple children, the survey oversampled for children with special health care needs, such that the child with these needs was more likely to be selected for the sample than their sibling. Results from the NSCH were weighted to reflect the greater population of non-institutionalized children and adolescents aged 0 to 17 years old, and all data are made publicly available.

The current study utilized three waves of data from 2016, 2017, and 2018. In 2016, 50,212 surveys were completed with a response rate of 40.7% (CAHMI, 2017). In 2017, 21,599 surveys were collected (37.4% response rate), and 30,530 surveys were collected in 2018 (response rate: 43.1%; CAHMI, 2019). The 2016 wave was analyzed separately from the 2017 and 2018 waves of data due to changes in survey wording. The two waves of data from 2017 and 2018 were combined into a single dataset (i.e., 2017/18 combined) in order to increase the sample size of adolescents with ASD, which more closely mirrors the sample size and demographic breakdown of the 2016 wave of data. Further, two datasets helped to foster reproducibility. The 2017/2018 combined dataset was provided by CAHMI and only includes survey items that were the same across the two years. Items weights were utilized to account for the combining of the 2017 and 2018 waves of data.

The current analysis used information reported by caregivers of adolescents who were in middle or high school (i.e. ages 12 to 17), mirroring the age segmentation of the NSCH survey. Our analyses examining sociodemographic disparities included only adolescents with a current diagnosis of ASD from either the 2016 or 2017/18 combined dataset in order to identify those individuals with ASD most at-risk of future social isolation and community disconnection.

### Measures

#### Autism Diagnosis.

Caregivers responded to the survey item, “*Has a doctor or other health care provider EVER told you that this child has Autism or Autism Spectrum Disorder (ASD)? Include diagnoses of Asperger’s Disorder or Pervasive Developmental Disorder (PDD)*.” Caregivers were also asked to indicate whether the diagnosis of Autism was current or past. Based on these responses, a variable was made available in the NSCH data set that indicated whether the child currently has Asperger’s Disorder, Pervasive Developmental Disorder or other Autism Spectrum Disorder. The response options included “*Does not have condition*”, “*Ever told but does not currently have condition*”, or “*Currently has condition*”. In our study, the available indicator variable was recoded into a dichotomous variable of “Has current autism spectrum disorder” or “Does not have autism spectrum disorder”. Those with the response of “Ever told but does not currently have condition” were excluded due to the lack of clarity about that child’s diagnosis. The current study included only those adolescents with a current diagnosis of ASD. Previous research has demonstrated validity of caregiver-reported ASD status (Daniels et al., 2012).

#### Extracurricular Participation in Activities.

Caregivers reported on their child’s involvement in three categories of after school activities: sports, clubs/organizations, and organized activities. Caregivers were asked, *“During the past 12 months, did this child participate in a sports team or did he or she take sports lesson after school or on weekends?”*. Caregivers were also asked, “*During the past 12 months, did this child participate in any clubs or organizations after school or on the weekends?”*. Caregivers were then asked, “*During the past 12 months, did this child participate in any other organized activities or lessons, such as music, dance, language, or other arts?”*. All three questions were coded as either “yes, the child did participate” or “no, the child did not participate”.

#### Community Involvement.

To assess community involvement, caregivers reported on whether their child had participated in work for pay or community service. Caregivers responded “yes” or “no” to the question, “*During the past 12 months, did this child participate in any paid work, including regular jobs as well as babysitting, cutting grass, or other occasional work?”* Caregivers also responded “yes” or “no” to the question, “*During the past 12 months, did this child participate in any type of community service or volunteer work at school, church [2016 wording]/place of worship [2017/18 wording], or in the community?”*

#### Child and Household Demographics.

Caregivers reported on their own education and household income, as well as their child’s sex. For caregiver report of child’s sex, responses were coded as male or female, as those were the only two options given. For household income, a composite variable was created in the NSCH dataset, which was comprised of information about the household’s combined income and household size. Household income was operationalized in relation to the federal poverty level (FPL), and it included four levels (i.e., 0–99% FPL, 100–199% FPL, 200–399% FPL, 400% or greater FPL). The caregiver completing the survey reported on the highest level of education they had completed, as well as the highest level of education the second primary caregiver of the child had completed. The highest level of education among the two primary caregivers was then made into a binary caregiver education variable (i.e. high school education or less vs. more than a high school education). Child and household demographics were examined as potential disparity indicators.

### Missing Data

In both the 2016 and 2017/18 combined datasets, missing data for sex was less than 1%, and hot-deck imputed values were used. Given that 22.83% of the weighted sample in 2016 and roughly 15% of the 2017/18 combined sample were missing information on household income in relation to the FPL, values were multiply imputed using sequential regression methods. In 2016, caregiver education was less than 1% and hot-deck imputation was used, while sequential regression imputation was utilized for missing values in 2017/18.

### Statistical Analyses

In both 2016 and 2017/18 combined, we used chi-square tests to identify significant differences among adolescents with ASD in their rates of participation in extracurricular activities (i.e., sports, clubs, organized activities) and community involvement (i.e., work for pay, community service) stratified by each child and household sociodemographic factor. To assess for age differences in rates of engagement in work for pay or community service, analyses were run among all adolescents with ASD, as well as among only high school aged adolescents with ASD (i.e., 14 to 17 years old). We additionally conducted logistic regression models to assess the adjusted main effects of each sociodemographic factor (i.e., sex, household income, caregiver education) on each extracurricular and community activity (i.e., sports, clubs, organized activities, work for pay, community service), controlling for the other sociodemographic factors. We further explored interactions between sociodemographic factors to determine whether the covariance of predictors contributed to participation. All analyses were conducted within each of the two datasets (i.e., 2016, 2017/18 combined) using SPSS Version 25 (IBM, 2016).

## Results

### Sample Characteristics

In 2016, there were 586 adolescents with ASD (mean age = 14.56 +/− 1.70, 81% male, 75% White). In 2017/18 combined, there were 716 adolescents with ASD (mean age = 14.60 +/− 1.62, 77% male, 71% White). In 2016, 50 adolescents were excluded, and, in 2017/18, 47 adolescents were excluded from these analyses because of diagnostic uncertainty (i.e., the caregiver responded that they were, “Ever told, but does not currently have condition [ASD]”. The sex distribution aligns with current prevalence estimates for ASD in males and females (Maenner et al., 2020). See Table 1 for demographic data.

### Extracurricular Participation: Adolescents with ASD

Participation rates in sports, clubs, and organized activities are in Table 2. Adjusted odds ratios for participation in sports, clubs, and organized activities are found in Table 4.

#### Sports.

**Figure 1.**
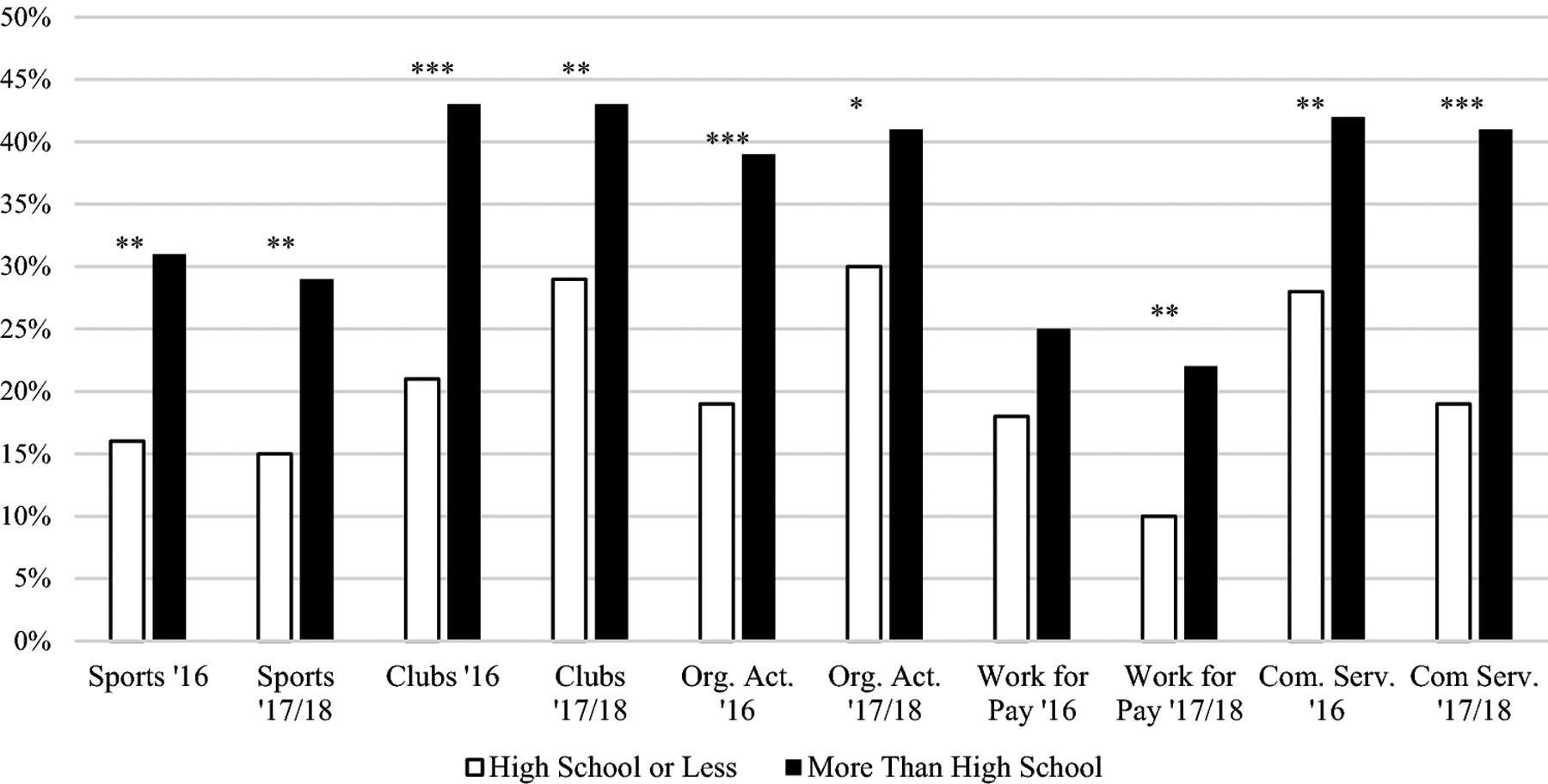
Percentage of youth with ASD who participated in sports, clubs, organized activities, work for pay, and community service in 2016 and 2017/18 stratified by caregiver education. Org. Act. = Organized Activities, Com. Serv. = Community Service. *p < .05, ** *p* < .01, ***p < .001

**Figure 2.**
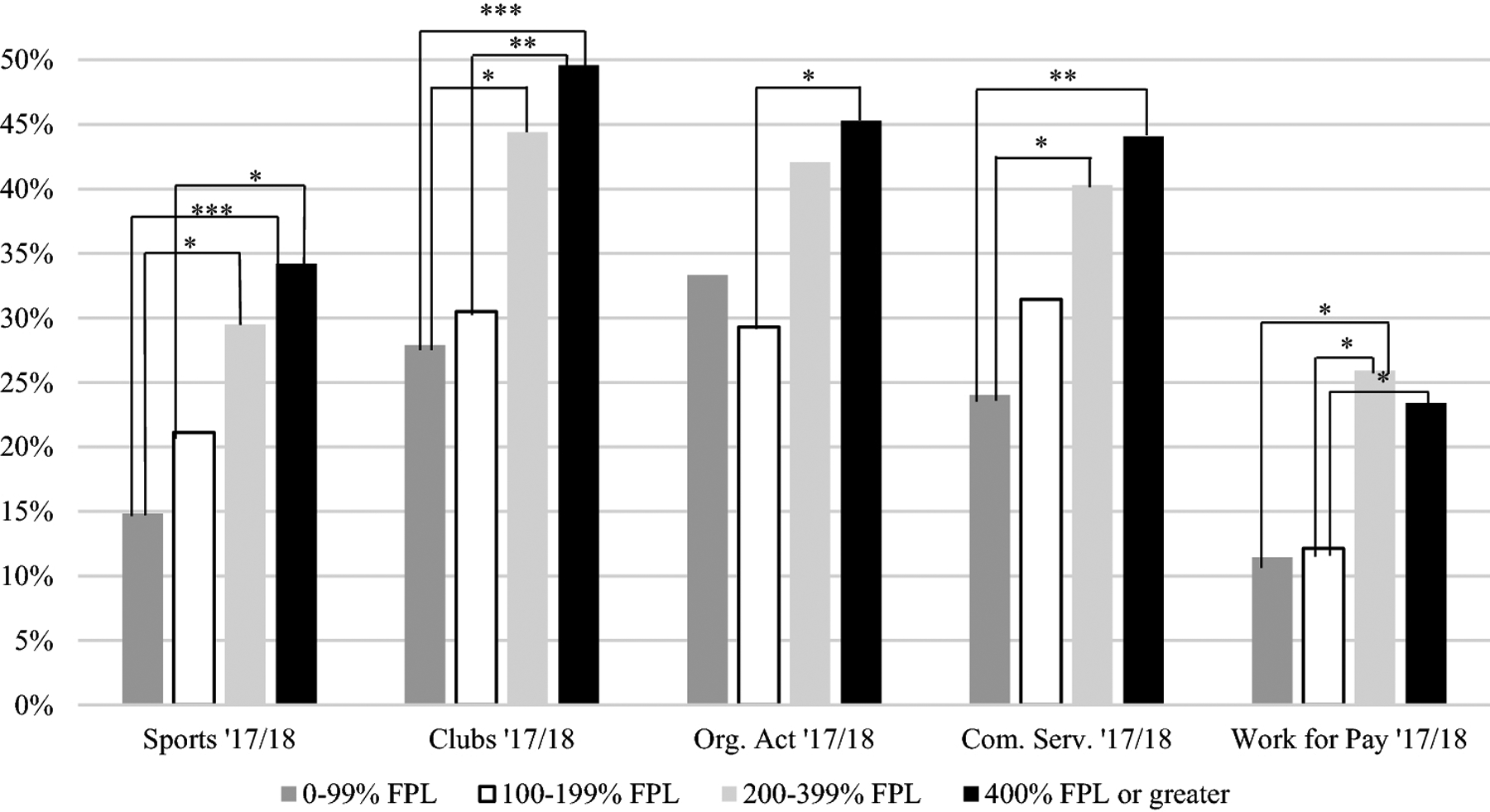
Percentage of youth with ASD in 2017/18 who participated in sports, clubs, organized activities, community service, and work for pay stratified by household income. FPL = Federal Poverty Level, Org. Act. = Organized Activities, Com. Serv. = Community Service. *p ≤ .05, ** *p* < .01, ***p < .001

Based on chi-square testing, in both 2016 and 2017/18, adolescents with ASD were less likely to be involved in sports if their caregiver reported an education level of high school or less compared to caregivers with more than a high school education (16% vs. 31%, *p* < .01 in 2016, 14% vs. 27%, *p* = .03 in 2017/18; Figure 1). In 2017/18 only, there were significant differences between the four levels of household income (*p* < .001; Figure 2). Adolescents from the highest income category (i.e., 400% or greater FPL) were more likely to engage in sports compared to adolescents in the lowest income category (i.e., 0–99% FPL; 34% vs. 15%, *p* < .001) and the second lowest income category (i.e., 100%−199% FPL; 34% vs. 21%; *p* = .04). Adolescents living in a household in the second highest income category (i.e., 200–399% FPL) had higher levels of engagement in sports compared to adolescents in the second lowest category (i.e., 100%−199%; 29% vs. 15%; *p* < .02). In 2016, there were no significant differences based on household income after adjusting for multiple comparisons.

The results from the multivariable regression model show that, in both 2016 and 2017/18, caregiver education was independently associated with engagement in sports, adjusting for all other factors. In 2017/18 only, household income was also independently associated with engagement in sports when controlling for all other sociodemographic factors. No significant interactions were found between the sociodemographic factors. No statistically significant differences were found for participation in sports based on sex.

#### Clubs.

**Figure 3.**
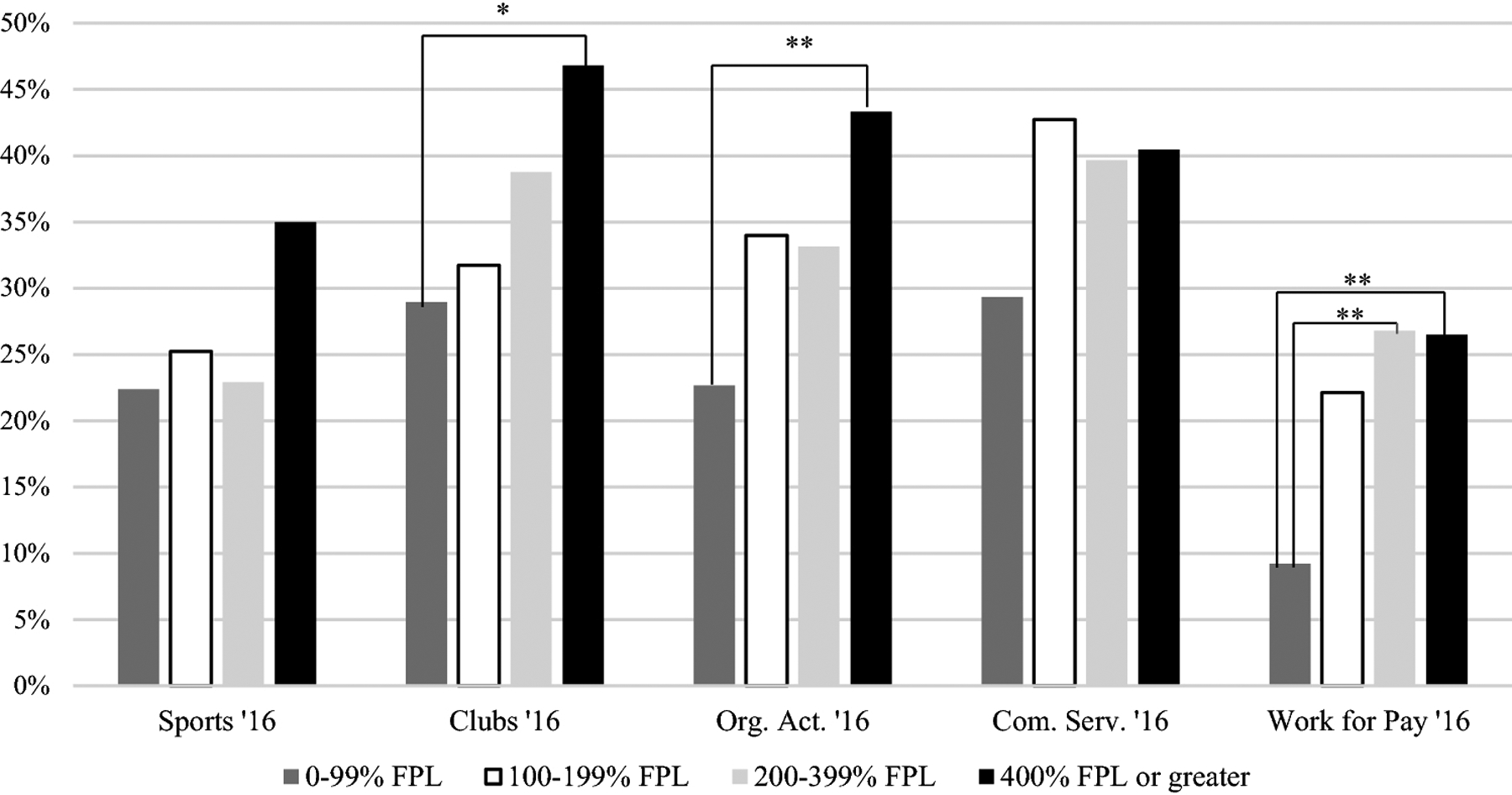
Percentage of youth with ASD in 2016 who participated in sports, clubs, organized activities, community service, and work for pay stratified by household income. FPL = Federal Poverty Level, Org. Act. = Organized Activities, Com. Serv. = Community Service. *p ≤ .05, ** *p* < .01, ***p < .001

Based on the chi-square results, in both 2016 and 2017/18, adolescents with ASD whose caregiver reported a high school education or less were less likely to be involved in clubs than those whose caregiver had more than a high school education (21% vs. 43%, *p* < .001 in 2016, 29% vs. 43%, *p* < .01 in 2017/18). In both 2016 and 2017/18, males with ASD were less likely to be engaged in clubs than females (37% vs. 48%, *p* =.03 in 2016, 39% vs. 48%, *p* = .03 in 2017/18). In 2016 and 2017/18, adolescents from the highest income category (i.e., 400% or greater FPL; Figure 3) were more likely to participate in clubs than those in the lowest income category (i.e., 0–99% FPL; 47% vs. 29% in 2016, *p* = .05, 50% vs. 28%, *p* < .001 in 2017/18). In 2017/18 only, adolescents from the second highest income category (i.e., 200–399% FPL) were more likely to participate than adolescents in the lowest income category (i.e., 0–99% FPL; 44% vs. 28%, *p* = .03). In 2017/18, adolescents in the highest income category (i.e., 400% or greater FPL) were more likely to participate than adolescents in the second lowest income category (i.e., 100%–199% FPL; 50% vs. 30%, *p* < .01).

In both 2016 and 2017/18, sex and household income were independently associated with participation in clubs when adjusting for all sociodemographic factors. In 2016 only, caregiver education was independently associated with engagement in clubs, adjusting for all other factors. No significant interactions were found between sociodemographic factors. No other statistically significant associations were found for participation in clubs across the remaining sociodemographic factors.

#### Organized Activities.

Based on chi-square testing, adolescents in both the 2016 and 2017/18 waves whose caregiver reported an education of high school or less were less likely to participate in organized activities compared to adolescents with caregivers reporting an education of more than high school (19% vs. 39%, *p* < .001 in 2016; 30% vs. 41%, *p* = .03 in 2017/18). In 2016 and 2017/18, males were less likely than females to be involved in organized activities (31% vs. 55%, *p* < . 001 in 2016, 37% vs. 48%, *p* = .02 in 2017/18). In 2016, adolescents from a household in the highest income category (i.e., 400% or greater FPL) were more likely to participate in organized activities compared to those in the lowest income category (i.e., 0–99% FPL; 43% vs. 23%, *p* < .01). In 2017/18, adolescents in the highest income (i.e., 400% or greater FPL) category were more likely to participate in organized activities than adolescents in the second lowest income category (i.e., 100%–199% FPL; 45% vs. 29%, *p* < .01).

In both 2016 and 2017/18, sex and household income were independently, significantly associated with rates of participation in organized activities when adjusting for all other sociodemographic factors. In 2016 only, caregiver education was independently associated with engagement in organized activities. No significant interactions were found.

### Community Involvement: Adolescents with ASD

Participation in community service and work for pay are displayed in Table 3. Adjusted odds ratios for participation in community service and work for pay are found in Table 4.

#### Community Service.

In both 2016 and 2017/18, results from chi-square testing show that adolescents with ASD had lower rates of participation in community service if they had a caregiver with a high school education or less (28% vs. 48%, *p* = .01 in 2016, 19% vs. 41%, *p* < .001 in 2017/18). In 2017/18 only, adolescents in the highest income category (i.e., 400% or greater FPL) and second highest income category (i.e., 200–399% FPL) were more likely to participate than adolescents in the lowest income category (i.e., 0–99% FPL; 44% vs. 24%, *p* < .01, 40% vs. 24%, *p* = .03, respectively). When exclusively examining high school aged adolescents, only the comparison between the highest and lowest SES category remained significant. All other findings for community service remained the same in the high school-aged adolescents only sample.

In 2016 and 2017/18, caregiver education was associated with engagement in community service, controlling for all other sociodemographic factors. In 2017/18 only, household income was significantly associated with community service, adjusting for all other factors. No interactions were found, and no other significant associations were found between involvement in community service and the sociodemographic factors examined.

#### Work for Pay.

Based on chi-square testing, in 2017/18 only, adolescents with ASD were less likely be involved in work for pay if they had a caregiver with an education level of high school or less compared to more than high school (10% vs. 22%, *p* < .01). In 2016 only, adolescents in the highest income category (i.e., 400% or greater FPL) engaged in work for pay more than adolescents in the lowest income category (i.e., 0–99% FPL; 26% vs. 9%, *p* = .01). In 2017/18 only, adolescents in the highest (i.e., 400% or greater FPL) and second highest income category (i.e., 200–399% FPL) engaged in work for pay more than adolescents in the second lowest income category (i.e., 100%–199% FPL; 23% vs. 12%, *p* = .05, 26% vs. 12%, *p* = .01, respectively). In both 2016 and 2017/18, adolescents with ASD in the second highest income category (i.e., 200–399% FPL) were more likely to work for pay compared to those in the lowest income category (i.e., 0–99% FPL; 27% vs. 9%, *p* = .01 in 2016, 26% vs. 11%, *p* = .02 in 2017/18). Across both 2016 and 2017/18, all associations remained the same for sociodemographic factors when examining high school-aged adolescents only.

In both 2016 and 2017/18, household income was independently associated with work for pay, controlling for all other factors. In 2017/18 only, caregiver education had a significant, independent association with work for pay, controlling for all other factors. No other significant associations were found between sociodemographic factors and work for pay, and there were no significant interactions between sociodemographic factors.

## Discussion

There are many social, emotional, physical, and vocational benefits of participation in extracurricular and community activities (Hilton, Crouch, & Israel, 2008; King et al., 2003; Murphy & Carbone, 2008). Despite these benefits, middle and high school adolescents with ASD are at risk for low levels of participation compared to typically developing adolescents and adolescents with other disabilities (Askari et al., 2015; Taheri et al., 2016; Shattuck et al., 2011). Among typically developing adolescents, participation and involvement are significantly lower for adolescents living in low SES households and rates for specific activities vary by sex (Im et al., 2016; Johnston, Delva, & O’Malley, 2007; Meier et al., 2018), yet these same factors previously showed mixed results among adolescents with ASD. By examining sociodemographic factors across multiple waves of NSCH data, we were able to 1) examine disparities among adolescents identified as having ASD using DSM-V criteria, 2) identify whether specific disparities replicated across years (given the limited sample sizes within year), and 3) examine consistency across multiple waves of the NSCH survey. The current study found that, among adolescents with ASD, there are significant disparities in extracurricular participation and community involvement based on sex, household income, and caregiver education.

### Extracurricular Activities

Consistent with sex differences observed in adolescents without ASD, our findings indicate that males with ASD were less likely to be involved in clubs or organized activities compared to females with ASD. This association remained significant after controlling for all other sociodemographic factors. Increasing engagement and access to accommodating clubs/organized activities for adolescent males with ASD will provide them with the opportunity to develop an identity, learn social expectations, and improve emotion regulation (Bohnert, Lieb, & Arola, 2019; Dovgan & Mazurek, 2019; Larson, Hansen, & Moneta, 2006; Denault & Poulin, 2009). In addition, participation in clubs and organized activities can help to establish friendships and connect these adolescents to a community of individuals who share similar interests, which may in turn buffer against social-isolation and lead to continued relationships post-high school (Pence & Dymond, 2015; Zambon et al., 2010).

In the current study, no sex differences were found for participation in sports, which may be due to overall low participation rates as a result of ASD-related challenges, such as motor and social difficulties, peer exclusion, or a limited number of accommodating sports programs (Menear & Neumeier, 2015; Vetri & Roccella, 2020). In prior research, typically developing adolescent males and females have similar rates of overall participation, with males more often engaging in sports and females more often engaging in clubs (Im et al., 2016). In this study, female adolescents with ASD are continuing to reap some of the benefits of participating in clubs and organized activities, while male adolescents with ASD are losing out on overall rates of participation given the reduction in both clubs/organized activities and sports participation. Future research should examine whether the influence of environmental barriers, such as peer rejection, staff training, or access to accommodating activities, differs across male and female adolescents with ASD.

Consistent with findings among typically developing youth (Im et al., 2016; Johnston, Delva, & O’Malley, 2007; Meier et al., 2018), the current study shows that adolescents with ASD whose caregiver reported lower educational attainment and lived in a lower income household were generally less likely to be involved in sports, clubs, and organized activities. These findings are consistent with previous research which identified that both cost and accessibility of activities are significant environmental barriers to participation (Egilson et al., 2017; Ghanouni et al., 2019; Krieger et al., 2018; Mattinson et al., 2018). It is also likely that there are mediating variables that were not examined in this study. For example, the neighborhoods in which these adolescents are living in may not have the resources to support individuals with ASD, or, if there are resources, these families may not be able to find them, navigate them, or afford them. Additionally, there may be insufficient outreach to engage families, or these families may feel unsafe or uncomfortable allowing their children to be involved in activities. On the other hand, these adolescents may be engaging in and reaping the benefits of activities that were not explored in this study. For example, these adolescents may spend more time with extended family members, and therefore, receive the benefit of strong familial relationships. Our findings suggest that increasing accessibility and outreach, while decreasing cost, may increase participation for families in a variety of living situations, yet this is an area for future study. The current study helps clarify previously mixed findings by demonstrating the independent influence of both caregiver education and household income on participation. These factors help to identify those adolescents with ASD who may be at greatest risk of low participation.

### Community Involvement

Engaging in the community is integral to the current functioning and future success for individuals with ASD. Unfortunately, adolescents with ASD continued to have diminished involvement in community activities, and these rates were significantly lower among adolescents whose caregiver reported a high school education or less. When examining caregiver education, we found that, in the 2017/18 combined sample only, adolescents with ASD whose caregiver reported a high school education or less were less likely to be involved in work for pay, and this association remained significant after controlling for other sociodemographic factors. In typically developing youth, if a caregiver reported lower educational attainment, there may be increased individual or familial pressure on the child to work during middle and high school (Mortimer, 2010). Lower rates of work for pay were observed in adolescents with ASD whose caregiver reporter lower educational attainment, even though these individuals may experience the same economic struggles as typically developing youth. This difference may be due to the fact that caregivers are not putting an additional emphasis on the need to work for their child with ASD, and/or it may be due to a lack of supportive employment opportunities.

When examining household income, in both 2016 and 2017/18, adolescents with ASD from lower income households were significantly less likely to engage in work for pay. This finding is consistent with studies looking at young adults with ASD, such that those young adults living in lower SES households had higher rates of unemployment compared to young adults with ASD living in higher SES households (Eilenberg et al., 2019). Our current study identifies that these SES-related disparities in work for pay are already present before young adulthood. The overall low rates of work for pay across all household income levels in the current study are particularly worrisome given that engaging in work during high school is integral to employment continuity as students transition out of school (Mortimer, 2010; Roux et al., 2015; Staff & Mortimer, 2007). Promoting engagement in vocational activities will require more accommodating work environments with trained staff, increased access to transportation, as well as social and school supports to assist youth with transitioning into workplace roles.

In both 2016 and 2017/18, adolescents whose caregiver reported lower educational attainment were less likely to be involved in community service. In 2017/18 only, adolescents from a household in the lower income category were less likely to engage in community service compared to adolescents from a higher income category. Both of these associations remained independently significant after adjusting for all other sociodemographic factors. No differences in community engagement were detected among adolescents based on sex, which may be due to overall low rates of engagement. Similar to our findings from above, it is possible that these adolescents are engaging in other activities or reaping other benefits that are not examined in the NSCH. Future research is needed to understand individual desires, as well as financial and familial pressure to engage in work for pay and community service, while taking into account the added challenges experienced by adolescents from disadvantaged backgrounds. When the sample was restricted to high school students only, the findings remained similar for work for pay and community service engagement.

### Limitations

There were limitations of the current study, which should be addressed in future research. First, the NSCH dataset relies on caregiver report of the child’s ASD status as indicated by a clinician, yet the diagnosis was not confirmed by a provider. Previous research demonstrates the validity of caregiver reported diagnosis of ASD (Daniels et al., 2012; Lee et al., 2010). While this information is easily collected for large surveys such as the NSCH, assessment of clinical severity and type of impairment is lost, which may differentially impact our findings. Second, previous studies have shown clear racial and ethnic disparities among typically developing youth in their rates of extracurricular participation, yet these same disparities have not been explored in a sample of adolescents with ASD. Unfortunately, our analyses based on race/ethnicity were uninformative and not included due to the small sample size and lack of diversity within the sample. Even though the NSCH is a large, national survey, diversity within mores specific race/ethnicity categories was small, especially within the Hispanic category. For example, among adolescents who identified as Hispanic, 74% identified as White in both 2016 and 2017/18. Despite the pressing need to explore disparities based on race/ethnicity, this dataset was not suitable to answer those questions. Future studies should oversample for a more diverse population of adolescents with ASD in order to examine whether disparities exist based on race/ethnicity. Interventions to increase participation should also take into account racial, ethnic, and cultural preferences and desires. Next, the frequency and duration of participation was not measured making it is difficult to assess whether adolescents obtained the known benefits of these activities. Future research should incorporate adolescent self-report to better understand the perceived benefits of participation, which may help elucidate the quality of participation. There may also be individual level factors contributing to decreased participation that were not captured, such as preferences to engage in other activities, ASD-specific challenges (e.g., emotional, behavioral, and social difficulties), co-occurring disorders (e.g., Intellectual Disability), as well as peer level factors, such as exclusion from teams and clubs. Targeted interventions to increase participation should focus on the multitude of contributing factors, including sociodemographic factors, individual- and peer-level factors, as well as previously identified barriers. Next, this study was cross-sectional and could not examine long-term outcomes, so longitudinal research is needed to assess whether participation in extracurricular activities and community involvement during middle and high school would help buffer against poor outcomes in young adulthood. Finally, this sample only included adolescents with ASD, so comparisons could not be made between adolescents with ASD and other populations. Future research should examine whether the sociodemographic disparities identified in the sample are similar among adolescents with other neurodevelopmental disorders.

### Implications

In order to begin closing these disparity gaps, efforts need to be taken to improve levels of participation and community involvement. A multi-faceted approach should incorporate caregivers, teachers, community stakeholders, and adolescents with ASD. Guiding caregivers on how to choose beneficial and appropriate activities and empowering adolescents with ASD to be active participants in deciding what activities best suit their interests and abilities is critical. In addition, it is necessary to work with teachers and adult leaders from the community in order to educate them about the specific supports an adolescent with ASD might require in order to participate (Grandisson et al., 2012; Krieger et al., 2018). By working with community and school leaders, efforts can be made to create or tailor activities to become more accessible, accommodating, and inclusive for adolescents with ASD. A family navigator or care coordinator (e.g., social worker) may be ideally suited to help stakeholders in this process (Myers et al., 2015). Existing programs may also need to improve outreach efforts in order to serve a more financially and racially diverse population of adolescents with ASD.

Further, targeted interventions should take into account preferred activities among subgroups of adolescents with ASD, in combination with identified disparities, in order to promote overall rates of participation. For example, this may focus on engaging adolescent males with ASD in clubs, organized activities, and sports. To buffer against the high rates of unemployment in young adulthood, targeted interventions should also focus on increasing inclusive work opportunities for adolescents with ASD, particularly those whose families have a lower income. Finally, additional vocational training and resources may be needed in these communities to help workplaces accommodate and support individuals with ASD. By increasing the number of employment and community service opportunities that are accessible and inclusive for adolescents with ASD during high school, the low rates and multiple disparities of community involvement seen during high school and in young adulthood may be diminished.

The disparities indicated in this paper are consistent with prior research showing widening disparities in access to diagnostic and therapeutic services (Bishop-Fitzpatrick & Kind, 2017; Karpur et al., 2019; Nguyen et al., 2016). Compared to other disabilities, youth with ASD of disadvantaged backgrounds have higher rates of unmet healthcare needs overall (Karpur et al., 2019). More specifically, youth with ASD who are not White are more likely to receive a delayed correct diagnosis, a longer time gap between diagnosis and treatment, and poorer health care access and quality compared to white youth (Bishop-Fitzpatrick & Kind, 2017). Additionally, in the United States, children and adolescents of a lower SES are more likely to receive fewer hours of treatment services (Nguyen et al., 2016). Among youth waiting for therapeutic services, engagement in extracurricular activities and community involvement may provide some benefits in the interim, such as learning social and communication skills, that may be expanded upon in more specialized interventions. Among youth currently receiving services, these activities may provide complementary skills and opportunities to generalize that are more difficult to achieve in a structured setting, such as naturalistic opportunities to learn social skills from peers.

### Conclusion

The current study used data from the three most recent waves of the National Survey of Children’s Health (2016, 2017, and 2018), and demonstrated that, among adolescents with ASD, participation in extracurricular activities and community involvement is lower among specific groups, based on sex, household income, and caregiver education. Given that young adults with ASD experience multiple poor outcomes, increasing engagement in extracurricular and community activities in middle and high school may buffer against these outcomes in young adulthood by establishing relationships, teaching vocational skills, and increasing academic engagement. Organization level interventions, such as appropriate training and accommodations, as well as individual level interventions, such as skills-based trainings, are needed to improve rates of engagement so that all adolescents with ASD, despite sociodemographic background, are able to reap the many benefits of community participation.

## Figures and Tables

**Table 1:** Child and Household Demographic Information for Adolescents with ASD

	2016	2017/18
	*n*=586	*n*=716
Child Race/Ethnicity	*n* (%)	*n* (%)
White, non-Hispanic	441 (75%)	509 (71%)
Black, non-Hispanic	36 (6%)	46 (7%)
Hispanic	49 (9%)	81 (11%)
Other, non-Hispanic	60 (10%)	80 (11%)
**Child Sex**		
Female	112 (19%)	164 (23%)
Male	474 (81%)	552 (77%)
**Household Income**		
0–99% FPL	76 (13%)	110 (15%)
100–199% FPL	105 (18%)	143 (20%)
200–399% FPL	181 (31%)	221 (31%)
400% or more FPL	224 (38%)	242 (34%)
**Caregiver Education**		
High School or Less	94 (16%)	114 (16%)
More than High School	477 (84%)	602 (84%)

*Note*. FPL = Federal Poverty Level.

**Table 2:** Extracurricular Participation: Sports, Clubs, and Organized Activities Among Youth with ASD

		Sports					Clubs						Organized Activities					
	2016			2017/18			2016			2017/18			2016			2017/18		
	*n*=586			*n*=716			*n*=586			*n*= 716			*n*=586			*n*= 716		
Child Sex	*n* (%)	*χ* ^2^	*p*	*n* (%)	*χ* ^2^	*p*	*n* (%)	*χ* ^2^	*P*	*n* (%)	*χ* ^2^	*p*	*n* (%)	*χ* ^2^	*p*	*n (%)*	*χ* ^2^	*p*
Female	25 (23%)	1.94	0.16	40 (25%)	.55	0.46	53 (48%)	4.94	0.03	77 (48%)	4.54	0.03	60 (55%)	21.7	< .001	76 (48%)	5.94	.02
Male	136 (29%)			151 (28%)			173 (37%)			207 (39%)			145 (31%)			196 (37%)		
**Household Income**																		
0–99% FPL	17 (22%)	9.26	.03	16 (15%)	17.44	.001	22 (29%)	10.77	.01	20 (28%)	22.04	.001	17 (23%)	11.66	.01	35 (33%)	11.67	.01
100–199% FPL	26 (25%)			30 (21%)			33 (32%)			43 (30%)			35 (34%)			41 (29%)		
200–399% FPL	41 (23%)			64 (29%)			69 (39%)			95 (44%)			59 (33%)			90 (42%)		
400% or More FPL	77 (35%)			81 (34%)			102 (47%)			117 (50%)			94 (43%)			106 (45%)		
**Caregiver Education**																		
High School or Less	15 (16%)	7.73	< .01	16 (15%)	9.79	< .01	19 (21%)	15.39	< .001	31 (29%)	6.99	<.01	17 (19%)	14.10	< .001	32 (30%)	4.63	0.03
More than High School	144 (31%)			175 (29%)			203 (43%)			253 (43%)			184 (39%)			240 (41%)		

*Note*. Number and percent of adolescents with ASD participating in each activity stratified by sociodemographic factors. FPL = Federal Poverty Level.

**Table 3:** Community Involvement: Work for Pay and Community Service Among Youth with ASD

	Work for Pay						Community Service					
	2016			2017/18			2016			2017/18		
	*n*=586			*n*=716			*n*=586			*n*= 716		
Child Sex	*n* (%)	*χ* ^2^	*p*	*n* (%)	*χ* ^2^	*p*	*n* (%)	*χ* ^2^	*p*	*n* (%)	*χ* ^2^	*p*
Female	31 (28%)	1.48	0.22	33 (20%)	.03	0.87	51 (47%)	3.29	0.07	64 (40%)	.62	0.43
Male	105 (22%)			107 (20%)			173 (37%)			196 (37%)		
**Household Income**												
0–99% FPL	7 (9%)	10.91	.01	12 (11%)	16.45	.001	22 (29%)	3.76	.29	25 (24%)	15.32	< .01
100–199% FPL	23 (22%)			17 (12%)			44 (42%)			44 (31%)		
200–399% FPL	48 (27%)			56 (26%)			71 (40%)			87 (40%)		
400% or More FPL	58 (26%)			55 (23%)			87 (40%)			104 (44%)		
**Caregiver Education**												
High School or Less	17 (18%)	1.59	0.21	10 (10%)	8.55	< .01	25 (28%)	6.53	0.01	20 (19%)	18.27	<.001
More than High School	116 (25%)			130 (22%)			197 (42%)			240 (41%)		

*Note*. Number and percent of adolescents with ASD participating in each activity stratified by sociodemographic factors FPL = Federal Poverty Level.

**Table 4:** Adjusted Odds Ratios for Extracurricular Participation and Community Participation

	Sports		Clubs		Organized Activities		Work for Pay		Community Service	
	2016	2017/18	2016	2017/18	2016	2017/18	2016	2017/18	2016	2017/18
	OR (95% CI)		OR (95% CI)		OR (95% CI)		OR (95% CI)		OR (95% CI)	
**Child Sex**	1.42 (.86–2.34)	1.20 (.80–1.81)	0.62* (.40–.96)	0.69* (.48–.99)	0.35*** (.22–.54)	0.65* (.45–.93)	0.76 (.47–1.23)	0.99 (.63–1.53)	0.67 (.43–1.03)	0.88 (.61–1.27)
**Household Income**	0.85 (.70–1.03)	0.74*** (.62–.88)	0.81* (.67–.96)	0.73*** (.63–.86)	0.79* (.65–.95)	0.83* (.71–.97)	0.78* (.64–.97)	0.78* (.64–.95)	0.97 (.81–1.16)	0.81** (.69–.94)
**Caregiver Education**	0.51* (.28–.94)	0.54* (.30–.97)	0.42** (.24–.73)	0.73 (.46–1.18)	0.41** (.23–.74)	0.74 (.46–1.18)	0.85 (.47–1.54)	0.47* (.23–.94)	0.54* (.32–.91)	0.41*** (.24–.70)

*Note*. Odds ratios for participation in each extracurricular and community activity adjusting for all other sociodemographic factors. OR = Odds Ratio. CI = Confidence Interval.

* *p* < .05,

** *p* < .01,

*** *p* < .001
